# “If I want to be able to keep going, I must be active.” Exploring older adults’ perspectives of remote physical activity supports: a mixed-methods study

**DOI:** 10.3389/fpubh.2024.1328492

**Published:** 2024-01-24

**Authors:** Samira Mehrabi, Sara Drisdelle, Hanna R Dutt, Laura E Middleton

**Affiliations:** ^1^Department of Kinesiology and Health Sciences, University of Waterloo, Waterloo, ON, Canada; ^2^Research Institute for Aging, Waterloo, ON, Canada

**Keywords:** physical activity, health promotion, older adults, home-based exercise, technology, virtual exercise, remote supports, mixed-methods

## Abstract

**Introduction:**

Pandemic-related public health restrictions limited older adults’ physical activity programs and opportunities. Physical activity supports shifted to remote options, however, information on their adoption and effectiveness is limited. This study aims to describe the remote supports received by older adults and their perceived effectiveness. Additionally, it aims to describe facilitators and barriers to remote supports for physical activity among older adults, particularly those reliant on technology.

**Methods:**

This study used an explanatory, sequential, mixed-methods design. Community-dwelling older adults (≥ 60 years) were recruited to partake in a web-based survey and an optional semi-structured follow-up interview informed by the COM-B model. Participant characteristics, perceived effectiveness of remote supports, and the presence and severity of barriers were described. Changes in physical activity levels before and during the pandemic were analyzed using the Wilcoxon signed-rank test. Qualitative data underwent inductive thematic analysis.

**Results:**

Fifty seven older adults (68.3 ± 7.1 years, 43 Female) completed the survey, of which 15 participants (67.4 ± 5.8 years, 12 Female) participated in interviews. The majority were Caucasian, highly educated, and resided in Canada. Total physical activity levels showed no statistically significant change from before to during the pandemic (*p* = 0.74); however, at-home exercise participation and technology usage increased. Pre-recorded and real-time virtual exercise supports were perceived as most effective. Main barriers included limited contact with exercise professionals, limited access to exercise equipment or space, and decreased mental wellness. Thematic analysis identified five main themes: (i) Enabled by knowledge and resources; (ii) Diverse motivations for physical activity; (iii) Fostering participation through social connection; (iv) Supervision and safety: enabling adherence; and (v) Virtual exercise: a sustainable option with technological considerations.

**Conclusion:**

Virtual platforms show promise in supporting older adults’ physical activity at home, especially for those with limited in-person access. Our study suggests that both real-time and pre-recorded virtual exercise supports are feasible, depending on technological capacity and support. While interactive real-time virtual programs allow interaction with professionals and peers, pre-recorded programs provide timing flexibility. Further research is needed to establish best practices for safe and effective virtual exercise programming, promoting its long-term adoption for supporting a wider range of older adults.

## Introduction

Physical inactivity and social isolation have major negative impacts on older adults’ health and well-being (1, 2). Physical inactivity is the fourth leading cause of death worldwide (3) and a major risk factor for many chronic conditions (4). Lack of physical activity is associated with adverse effects on older adult’s physical and mental health, functional independence, and quality of life (1). Social isolation and loneliness further compound negative health outcomes associated with physical inactivity as it increases the risk of depression and anxiety, as well as cognitive decline and dementia in older adults (5, 6). Socially isolated older adults tend to be less physically active and are at higher risk of falls and hospitalization (7). The World Health Organization (WHO) recommends older adults to engage in a minimum of 150 min of moderate intensity or 75 min of vigorous intensity physical activity per week, including both aerobic and strengthening exercises (8). Similarly, the Canadian Society for Exercise Physiology (CSEP) physical activity guidelines recommends accumulating 150 min of moderate-to-vigorous aerobic physical activity per week for individuals aged 65 and older, along with muscle-strengthening activities at least 2 days per week and balance exercises regularly (9). Despite the existing guidelines and evidence on the physical and mental benefits of physical activity (10, 11), physical inactivity increases with advancing age (12–14). In Canada, 87% of older adults are not sufficiently active to meet the guidelines of 150 min/week of moderate-to-vigorous intensity physical activity (15).

Physical inactivity and social isolation in older adults were exacerbated with public health restrictions in response to the novel coronavirus (COVID-19) pandemic (16, 17). With the rapid spread of the virus across the globe, local and federal governments enacted public health restrictions to contain the transmission of the virus (18). Older adults often faced more severe public health restrictions than other populations, due to their higher vulnerability for negative outcomes (19, 20). While these measures were crucial in preventing COVID-19 infections and lowering mortality rates, they also had adverse physical and psychosocial impacts on older adults, such as decreased social interaction and restricted access to facilities and programs, including those related to physical activity (21, 22). As a result, opportunities for physical activity among older adults were severely limited during the pandemic, with observed declines in both incidental and structured physical activity levels (23, 24). Furthermore, the disruption caused by the pandemic to community-based exercise programs resulted in a notable reduction in physical activity among older adults who were previously engaged in group-based exercises (25).

With the increasing prevalence of sedentary lifestyles among older adults, compounded by the social distancing measures of the COVID-19 pandemic, promoting home-based physical activity became more important. To support positive health behaviors and overall well-being during the pandemic, many exercise professionals transitioned to providing remote support for physical activity in lieu of traditional onsite programming. They used technology-based strategies, such as phone or text reminders, emails, and pre-recorded or real-time virtual exercise programming to assist their clients, including older adults (26–30). Pre-pandemic research has shown promising outcomes using technologies such as video-conferencing platforms for the delivery of health-related services, primarily rehabilitation (31). Despite the growing interest in promoting home-based physical activity among older adults through technology, there is limited research on the barriers and facilitators to, as well as the perceived effectiveness of, such supports for physical activity. Understanding these factors will be crucial in informing the design and implementation of safe and effective remote supports for older adults’ physical activity when in-person programs and facilities are not accessible. Therefore, we aimed to: (i) describe remote supports for physical activity received by older adults during the first year of the COVID-19 pandemic and their perceived effectiveness; and (ii) describe facilitators and barriers to adopting remote supports for physical activity among older adults during the COVID-19 pandemic and beyond, with a focus on technology-based supports.

## Materials and methods

### Study design

This study used an explanatory, sequential, mixed-methods design, incorporating both quantitative and qualitative data collection methods. Qualitative and quantitative methods were given equal weight, and their findings were integrated for interpretation using a contiguous approach, meaning that the results are presented in a single report with the qualitative and quantitative findings reported separately (32).

The data collection process started with the administration of a cross-sectional web-based survey to collect quantitative data, followed by semi-structured one-on-one interviews to collect qualitative data. The online survey was open from June 2020 to September 2020, and interviews were conducted between September 2020 to December 2020.

### Study sample and recruitment

To be eligible, participants were required to be: (i) aged ≥60 years; (ii) living in a community setting; (iii) able to communicate in English; and (iv) able to have access to an electronic device (computer, tablet, smartphone) and internet connection. There were no specific exclusion criteria; however, naturally participants would be unable to complete the web-based survey if they did not have access to the technology or internet required for participation. A combination of convenience and snowball sampling was used to recruit participants. Recruitment was done using social media platforms such as Facebook, LinkedIn, and Twitter, along with word-of-mouth. Additionally, local organizations that served older adults, such as senior centers or community centers, promoted the study via email lists and newsletters. There was no monetary compensation provided to participants for their participation in the study.

### Web-based survey

Participants completed the survey using the Qualtrics Survey platform [Qualtrics XM, Provo, UT] (33). The survey included questions to assess participants’ demographics (age, gender, ethnicity, highest level of education, country of residence, and living arrangements) and health (self-rated physical and mental health, self-reported chronic conditions, number of falls in the past 12 months, and use of mobility aids). Information regarding participants’ physical activity before and during the first year of the COVID-19 pandemic was collected using an adapted version of the Physical Activity Scale for the Elderly (PASE) (34). PASE is a validated questionnaire to assess older adults’ physical activity over the past 7 days (34). To make PASE more applicable to the context of this study, reporting of the frequency and duration of exercise was changed from “*over the past 7 days*” to “*in a typical week*” before and during the COVID-19 pandemic. For instance, instead of asking: “*Over the past 7 days, how often did you take a walk outside your home or yard for any reason?*,” participants were asked: “*In a typical week before the COVID-19 pandemic, on average, how often did you take a walk outside your home or yard for any reason*?.” The same structure was used to adapt PASE questions regarding older adults’ physical activity behaviors during the COVID-19 pandemic (i.e., “*Currently, in a typical week, on average, how often do you take a walk outside your home or yard for any reason*”). Additionally, the survey inquired about the participants’ at-home and outdoor exercise activities before and during the pandemic, as well as the frequency of their participation in facility-based (before the pandemic) and web-based (during the pandemic) exercise classes. To answer these questions, participants were asked to select from the following options: *“Never,” “Less than weekly,” “1–2 times per week,” and “3 or more times per week.”*

Further questions were asked concerning the type of remote supports for physical activity received by older adults including: (i) exercise programs and/or instructions received via mail (hard copy) or e-mail; (ii) check-ins or instructions via phone or web-chat; (iii) pre-recorded and real-time virtual exercise programs and/or videos; and (iv) access to resources on how to stay active at home (e.g., informational websites). Participants were subsequently asked to rate the perceived effectiveness of received supports using a 4-point Likert scale ranging from “*not effective at all*” to “*very effective*.” Similarly, participants were prompted to rate the perceived barriers to adopting remote supports for physical activity using a 5-point Likert scale ranging from “*not at all limiting*” to “*extremely limiting*.” Lastly, the survey delved into participants’ access to technology at home (including smart physical activity trackers) and their familiarity with and utilization of technology during the COVID-19 closures.

### Semi-structured interviews

Following completion of the survey, email invitations were sent to all survey respondents who expressed willingness to participate in a semi-structured interview. Since data collection took place during the COVID-19 pandemic, all interviews were conducted remotely via phone or a video conferencing platform, depending on the participant’s preference. The interview script was guided by the COM-B model and its three key elements (i.e., capability, opportunity, and motivation) (35). The interview questions and follow-up probes were designed to be open-ended and aimed at obtaining in-depth information about: (i) the physical activity experiences of older adults before and during the pandemic; (ii) their adoption and utilization of technology during the pandemic; (iii) the uptake and perceived effectiveness of remote physical activity supports; and (iv) facilitators and barriers to adopting remote supports for physical activity both during the pandemic and into the future, particularly those supports reliant on technology.

### Data analysis

#### Quantitative

Given the exploratory nature of this study, no formal sample size calculation was performed. Descriptive statistics were calculated for participant characteristics and were presented as means and standard deviation (SDs) or percentages [*n* (%)] as appropriate. Statistical analyses were performed in RStudio [R Foundation for Statistical Computing, Version 1.3.1093].

A Wilcoxon signed-rank test was used to explore within-group changes in physical activity among older adults from before the pandemic to during the pandemic. Additionally, descriptive statistics were used to characterize the perceived effectiveness of remote physical activity supports and presence and severity of barriers.

#### Qualitative

We embraced a post-positivist approach, acknowledging the existence of a single, objective reality that can be comprehended to a certain extent through empirical observations and rigorous scientific methods (36, 37). While we made a conscious effort to maintain objectivity throughout the process of data collection and analysis, we also recognized that our understanding and interpretation of participants’ experiences could potentially be influenced by our personal values, biases, assumptions, as well as our academic and non-academic experiences (36, 37).

All interviews, whether conducted over the phone or via video-conferencing, were digitally recorded. A trained research assistant manually transcribed all interviews verbatim (mean interview length of 42.5 ± 13 min). The transcripts underwent a thorough double-checking process by the lead author to ensure accuracy. The transcripts were cleaned, anonymized, and uploaded into NVivo (version 13; QSR International) (38) for analysis. To identify key topics and patterns of meaning across the interviews, the lead author (SM) and three research assistants familiar with the study conducted an inductive thematic analysis using Braun and Clarke’s 6-phase framework (39). First, they familiarized themselves with the depth and breadth of the data through repeated and detailed readings of the transcribed interviews, followed by independent and line-by-line coding. The preliminary codebook underwent multiple iterations to ensure its comprehensiveness and accuracy in capturing the essence of existing data. Next, relevant codes were collated and sorted into initial themes and sub-themes. Preliminary themes and subthemes were iteratively refined, in consultation with the senior researcher (LEM), until the themes were coherent, meaningful, and clearly distinct (39, 40). Themes without enough supporting data or those with too diverse content were revised, merged with other themes, or discarded. Lastly, themes and sub-themes were labeled, and a detailed description of their essence was generated, including quotes as examples of raw data (39).

The following measures were taken to ensure the rigor and trustworthiness of the study (36, 40–43). The team met frequently throughout the analysis process to debrief, review, and refine the codebooks and emerging themes/sub-themes, and to resolve any major analysis discrepancies or challenges. An audit trail was also established by preserving the audio recordings of the interviews, along with field notes and debriefing notes, and through reflexive memoing throughout the study. Additionally, all stages of the study were clearly documented and described in detail, including in-depth descriptions of the research methods, the setting, and the data collected, as well as a comprehensive and thorough account of the research findings.

## Results

### Participants

Fifty-seven older adults completed the survey with an average age of 68.3 ± 7.1 years. The majority were Caucasian (79%), identified as female (75%), had a post-secondary diploma or degree (83%), and had very good-to-excellent self-rated physical health (58%) and mental health (60%). Thirty-one older adults initially expressed interest in the interview, of which fifteen agreed to participate (9 via phone, 6 via Zoom). The average age of interviewees was 67.4 ± 5.8 years, with 87% identifying as Caucasian, 80% as female, and all possessing a post-secondary diploma or degree. Participant characteristics are presented in Table 1.

**Table 1:** Participant characteristics [mean (SD) or % (*n*)].

Characteristic	Survey (*n* = 57)	Interview (*n* = 15)
Age, years	68.3 (±7.1)	67.4 (±5.8)
Gender, female	75.4% (43)	80.0% (12)
Male	22.8% (13)	20.0% (3)
Prefer not to disclose	1.8% (1)	0.0% (0)
Ethnicity, Caucasian	78.9% (45)	86.6% (13)
South Asian	3.5% (2)	0.0% (0)
Indigenous	1.8% (1)	6.7% (1)
Prefer not to disclose	15.8% (9)	6.7% (1)
Country of residence, Canada	92.9% (53)	100% (15)
United States	5.3% (3)	0.0% (0)
United Kingdom	1.8% (1)	0.0% (0)
Highest level of education, ≥ Post-secondary degree	82.5% (47)	100% (15)
≤ High school diploma or equivalent	17.5% (10)	0.0% (0)
Self-rated physical health, ≥ Very Good	57.9% (33)	80.0% (12)
Good	28.0% (16)	20.0% (3)
Fair	12.3% (7)	0.0% (0)
Poor	1.8% (1)	0.0% (0)
Self-rated mental health, ≥ Very Good	59.7% (34)	53.3% (8)
Good	28.0% (16)	33.3% (5)
Fair	12.3% (7)	13.3% (2)
Poor	0.0% (0)	0.0% (0)

### Quantitative results

#### Physical activity participation and technology use during the COVID-19 pandemic

There were no statistically significant differences in overall physical activity levels reported during the first year of the pandemic (modified PASE score = 107.04 ± 71.06) compared to those reported during the pre-pandemic period (modified PASE score = 103.95 ± 62.78, *p* = 0.74). However, substantial variability was observed in the physical activity scores before and during the pandemic. Among participants, 43% reported an increase in their physical activity levels during the pandemic, while 11% maintained their activity levels and 46% experienced a decrease. Before the pandemic, more than half of participants (57%) took part in an onsite group-based exercise program at least once a week, with nearly one third (31%) attending these in-person programs three or more times per week. During the pandemic, over half of participants (51%) engaged in virtual group-based exercise classes. The number of participants engaged in at-home exercise at least once a week increased from 37% pre-pandemic to 70% during the first year of the pandemic.

When asked about the availability and usage of technology at home, 94% of participants indicated having access to a computer (e.g., desktop or laptop) and 87% had access to a mobile device (e.g., tablets, smart phones, etc.). Additionally, nearly half of the participants (45%) reported having access to smart physical activity trackers such as smartwatches or smartphone apps. Furthermore, 47% of participants noted an increase in their technology usage during the pandemic, while 39% acquired new technological skills to better support their physical activity behaviors.

#### Received remote supports for physical activity and their perceived effectiveness

The most common means of support received by older adults were real-time virtual exercise programs (received by 44%) and pre-recorded exercise videos (received by 35%). Real-time virtual exercise programs were perceived as being the most effective support for at-home exercise, with 92% of older adults rating it as “*moderately effective”* or “*very effective.”* Pre-recorded exercise videos were also rated to be highly effective supports, ranking as “*moderately effective”* or “*very effective”* by 75% of participants. While only a small proportion of participants (9%) received phone or web-chat check-ins, the majority of those who received check-ins perceived them to be *“moderately effective”* or *“very effective”* (80%). Table 2 provides a summary of the remote physical activity supports received by our participants and their perceived effectiveness.

**Table 2:** Remote physical activity supports and their perceived effectiveness [% (*n*)].

Physical activity supports		Perceived effectiveness			
	Received by participants	Very effective	Moderately effective	Slightly effective	Not effective
Real-time virtual exercise programs	43.9% (25)	72.0% (18)	20.0% (5)	0% (0)	8.0% (2)
Pre-recorded exercise videos	35.1% (20)	50.0% (10)	25.0% (5)	15.0% (3)	10.0% (2)
E-mailed exercise programs/instruction	24.6% (14)	28.6% (4)	7.1% (1)	35.7% (5)	28.6% (4)
Information for at-home exercise	22.8% (13)	7.7% (1)	15.4% (2)	46.2% (6)	30.8% (4)
Phone/web-chat check-ins	8.8% (5)	40.0% (2)	40.0% (2)	0% (0)	20.0% (1)
Phone/web-chat instruction	8.8% (5)	0% (0)	40.0% (2)	20.0% (1)	40.0% (2)
Hardcopy exercise programs/instruction	1.8% (1)	100% (1)	0% (0)	0% (0)	0% (0)

#### Perceived barriers to the adoption of remote supports for physical activity

Many survey respondents did not identify any significant barriers that *“extremely”* restricted their physical activity; however, lack of contact with exercise professionals was perceived to be the most limiting factor (19% reported as *“very limiting”* or *“extremely limiting”*), followed by a lack of access to equipment or space to exercise (17% reported as *“very limiting”* or *“extremely limiting”*). While fewer respondents reported mental health as a *“very limiting”* or *“extremely limiting”* factor, nearly half (47%) reported it to be at least “*slightly limiting.”* Table 3 presents a summary of the perceived barriers to adopting remote physical activity supports during the first year of the pandemic.

**Table 3:** Perceived barriers to remote physical activity supports [% (*n*)].

Perceived barriers	Not limiting	Slightly limiting	Moderately limiting	Very limiting	Extremely limiting
Decreased mental wellness	52.6% (30)	17.5% (10)	14.0% (8)	8.9% (5)	7.0% (4)
Lack of access to equipment or space	54.4% (31)	21.0% (12)	7.0% (4)	12.3% (7)	5.3% (3)
Lack of contact with exercise professionals	54.4% (31)	10.5% (6)	15.8% (9)	7.0% (4)	12.3% (7)
Lack of family/peer support	54.4% (31)	19.3% (11)	14.0% (8)	12.3% (7)	0% (0)
Competing demands	59.6% (34)	19.3% (11)	14.0% (8)	5.3% (3)	1.8% (1)
Lack of exercise knowledge	68.4% (39)	12.3% (7)	12.3% (7)	3.5% (2)	3.5% (2)
Lack of technological skills	70.1% (40)	17.5% (10)	5.3% (3)	5.3% (3)	1.8% (1)
Fear of injury	73.6% (42)	8.8% (5)	10.5% (6)	1.8% (1)	5.3% (3)
Safety concerns	75.4% (43)	8.8% (5)	8.8% (5)	3.5% (2)	3.5% (2)
Lack of technological resources	89.4% (51)	5.3% (3)	1.8% (1)	3.5% (2)	0% (0)

### Qualitative results

Five main themes emerged from our thematic analysis: (i) Enabled by knowledge and resources; (ii) Diverse motivations for physical activity; (iii) Fostering participation through social connection; (iv) Supervision and safety: enabling adherence and (v) Virtual exercise: a sustainable option with technological considerations.

#### Theme 1: enabled by knowledge and resources

The physical activity levels of participants during the pandemic were significantly influenced by their exercise knowledge, resources, and opportunities at and around their home. Participants’ prior experience with physical activity, as well as the availability of equipment and space in and outside their homes, played crucial roles in determining the uptake and maintenance of physical activity and exercise during this time.

(i) Prior exercise knowledge and skills

Many participants noted that they were able to stay active due to their exercise knowledge, skills and/or experience from before the pandemic. Established physical activity habits motivated some participants to continue engaging in at-home exercise despite the closure of fitness facilities: “*I’ve just always been fit and interested. I have done all of it [exercise] for a long time, so it’s been easy to continue doing it and I reaped the benefit of it.*” *(Female, 71 years old).* Participants’ knowledge, skills, and experience also played a crucial role in fostering a sense of safety and reducing the perceived risk of injury during home-based exercises. Prior exercise experiences enabled some participants to recognize their limits and feel confident to engage in unsupervised physical activity, as one participant described: “*I would not push myself if I felt like “this is the limit,” I do not need to feel sore or get any injuries.*” *(Female, 60 years old).* Others made reasoned choices to reduce the intensity of their exercise at home to minimize the risk of injury:

*But at home, I recognized that I was kind of vulnerable so I would decrease the amount of weight that I was using and just do more repetitions...so there was less chance of me hurting myself because the weights I was using were lighter. (Female, 60 years old).*

(ii) Adapting resources and space

Adequacy of exercise resources (including exercise equipment and access to reliable internet) and space at home emerged as factors that impacted older adults’ ability to engage in home-based exercise during the pandemic. Some participants adapted well to the available space within their homes: “*I’ve got a small apartment, but I have enough space, you do not need a lot of space for this [exercise], so that’s one good thing.*” *(Female, 72 years old);* whereas others expressed that the limitations of their home environment made it difficult to exercise: “*The one thing that could be a challenge would be just the proper space. We live in a condo at this point and are exercising and being aware of the noise that we could be making.*” *(Female, 74 years old).* Some mentioned using exercise equipment that they already owned, whereas others purchased new equipment (e.g., resistance bands) to support their at-home exercise routine, having additional time to partake in new activities due to the pandemic. Beyond exercise equipment, having access to a reliable internet connection was also deemed to be vital for participating in real-time virtual exercise classes. One participant noted: “*I’m lucky, my internet does not cut out, where some of the others - even the instructors, there’s times when we are doing exercise all at once, their screen will freeze.” (Female, 78 years old).*

(iii) Outdoor environment: an opportunity and a barrier

The physical environment for older adults was identified as both a significant motivator and barrier to physical activity during the pandemic. Access to parks and trails provided opportunities for community walking, which was identified by many as an enjoyable activity. Additionally, as exercise facilities remained closed over the summer of 2020, the warmer weather offered alternative outdoor options such as walking and gardening, motivating some older adults to become or stay physically active. For instance, one participant shared: *“With summer I love gardening... at first my husband and I were going for walks, and then once gardening started I kind of saw my exercise as being gardening instead of walking.” (Female, 79 years old).* Conversely, participants commonly noted that winter weather limited their outdoor physical activity, especially in the early months of the pandemic. This limitation was primarily due to safety concerns linked to engaging in outdoor physical activity in cold or icy conditions, as explained by one participant: “*I walked quite a bit if the weather was nice, but I do not walk in the winter, I do not wanna fall down. Weather wise I just will not [walk] when it’s icy so that was limiting.” (Female, 72 years old).*

#### Theme 2: diverse motivations for physical activity

Older adults expressed a range of factors that motivated them to stay physically active during the pandemic. Improving or maintaining physical and mental health was often described as a major intrinsic motivator for physical activity during the pandemic. Participants recognized the importance of staying physically active to support their overall health, including enhancing functional abilities and maintaining independence. For instance, one participant stated: *“I live by myself, so I must have focus that keeps me going so I do not become a burden for my family. It’s important that I look after myself. They do not need to be worried about me.” (Female, 78 years old).* Physical activity was also seen as a strategy to alleviate existing health conditions, such as arthritis, which motivated some older adults to exercise despite the challenges. As one participant shared:

*I’m open to anything because that’s what’s keeping me going; I have arthritis, so my knees can give me a lot of trouble. It hurts to do nothing, it hurts to do something, {laughs} so if I want to be able to keep going, I must be active. Must. (Female, 78 years old).*

Supporting one’s overall mental health also emerged as a prominent motivator for older adults to remain physically active during the pandemic. Although the connection between mental health benefits and engagement in physical activity varied across participants, enhancing mood appeared to be a primary intrinsic motivator for many. One participant highlighted: “*For me, the biggest thing is that I feel much happier, much better when I’m done exercising, when I’ve done my bit for the day and when I’ve accomplished something. That’s the biggest thing.*” *(Male, 63 years old)*.

#### Theme 3: fostering participation through social connection

Connecting with family, friends, and peers played a vital role in supporting older adults to maintain their physical activity levels during the pandemic. This was especially evident through the utilization of real-time virtual exercise classes, which not only facilitated social interactions but also enhanced engagement in physical activities.

(i) Encouragement from family, peers, and community members

The support of others was commonly credited for building and maintaining physical activity engagement during the lockdown periods. For some older adults, physical activity was already a significant part of their family’s routine before the pandemic. For others, it quickly became a new routine during the pandemic, creating an opportunity to engage in meaningful activities alongside their family members: “*My husband became a workout partner for the first time in our lives...we would literally head to the basement together for our workouts every morning during the lockdown.*” *(Female, 64 years old).* Community members also played a significant role in motivating older adults to engage in physical activity. For instance, one participant emphasized the influential impact of peer support in facilitating their exercise participation: *“A friend was doing the [exercise] program with me… we would contact each other every day, and it kind of provided a buddy system for each other.” (Female, 74 years old).*

On the other hand, participants who were usually motivated to exercise due to in-person social support and connections faced challenges when attempting to exercise independently. One participant expressed this struggle, saying: “*Because my activities were intertwined with my social life, it has been hard not having the motivation of my peers and my [rowing] crewmates to do these things.” (Female, 64 years old).* Similarly, another participant mentioned: “*I hate to admit it, but I just do not have the motivation to exercise by myself.*”*(Female, 68 years old).*

(ii) Social support with interactive virtual exercise classes

Although participants acknowledged and valued all remote physical activity supports that they received, interactive real-time virtual exercise classes emerged as the preferred option for most. These classes offered one of the few opportunities for social contact during the pandemic, making them particularly appealing. As one participant expressed: “*I like the live programs because that gives you the social feedback...you feel part of a group, which you are missing out on right now with the COVID.*” *(Female, 72 years old).* Interacting with peers in these exercise classes increased enjoyment and helped to create a sense of normality among participants: “*This morning we did a virtual fitness class. You see people you know in your neighborhood, and you can say hi and that sort of thing, it kinda makes it [virtual exercise] a little more normal.*” *(Male, 67 years old).* Such interactions also fostered a sense of accountability toward others, increasing their exercise: “*After my mom died, I found that I really do better with a schedule, or with other people, something that keeps me coming back.” (Female, 79 years old).*

Real-time virtual exercise classes also offered an opportunity for interaction between participants and exercise professionals, which was widely discussed as a significant benefit. Participants often described how exercise providers made the class more enjoyable: *“Some of our leaders will push us in a nice way. [Exercise providers] will push us because we can become lazy {laughs}, so they challenge us, and some days it’s hard work.” (Female, 78 years old).* In contrast, remote supports lacking social interaction, such as hard copy exercise programs, were considered relatively ineffective by some participants, many of whom only started to engage in at-home exercise when more interactive options became available. A participant shared: “*At first, the city sent us [hardcopy] exercises that told us what we could do, but I was never quite motivated to do them…but once they began the [virtual exercise] meetings I got started with it.” (Female, 79 years old).*

#### Theme 4: supervision and safety: enabling adherence

Safety remained a concern for some older adults when exercising at home, particularly in comparison to the perceived safety of gyms or community centers. Older adults noted that interactive virtual exercise classes felt safer compared to less-interactive options, as real-time interaction enabled virtual supervision by exercise professionals and fostered a sense of security for home-based exercise:

*The thing that I really like is that I can really feel a sense that everything they [exercise providers] are doing, to make sure that we are safe and we do not injure ourselves and, in case something was to happen, we’d have instructions as to what we can do. (Female, 79 years old).*

Exercise providers were often described as highly attentive to participant’s safety, with one older adult recalling the use of individualized reminders by their exercise provider to help reduce the risk of injury when exercising:

*I know they are always watching me, and they always tell you first thing to “put on your running shoes.” I wear bare feet all the time so sometimes I need a reminder to wear running shoes. So, I feel completely safe and that’s one thing they put right at the top, is the safety of it. (Female, 72 years old).*

Although interactive virtual exercise opportunities were greatly appreciated by most participants, for some, these options did not replace in-person guidance:

*I really liked in-person support, so if I’m doing weight resistance, I know that I’m doing it properly. As opposed to watching somebody on a stream, you do not know if your posture is correct or if you are doing it right. (Female, 68 years old).*

#### Theme 5: virtual exercise: a sustainable option with technological considerations

Participants viewed virtual exercise programs, including both real-time and pre-recorded, as a suitable support for physical activity beyond the COVID-19 pandemic. Such programming helped older adults to overcome some barriers to physical activity; however, the extent of its adoption was significantly shaped by individuals’ comfort levels with technology.

Virtual exercise classes were widely accepted as being an effective support among older adults during the pandemic and several participants expressed willingness to integrate virtual options into their regular exercise routine following the pandemic. Unforeseen circumstances unrelated to the pandemic, such as inclement weather, scheduling conflicts, and lack of access to transportation, were some of the driving factors that would encourage older adults to continue with virtual exercise programs in the future. As one participant shared: “*I would be quite ready and willing and wanting to do the virtual [exercise] if I was not able to go [in-person] because of weather.*” *(Female, 63 years old).* Some participants also noted that virtual exercise classes may better accommodate their schedule compared to in-person programming: *“I can do it whenever I feel like it. I’m not restricted to a particular time cause I’m not in a class.” (Female, 67 years old).*

Interactive real-time virtual classes were preferred by individuals who valued socialization as a key exercise motivator. However, pre-recorded exercise videos provided a unique benefit of flexible scheduling for others. Many participants valued the freedom to select class times, as one individual explained: “*I enrolled in an online yoga class, so I could, at whatever time of the day, log on and do yoga for an hour, which I was pretty faithful to.*” *(Female, 66 years old)*. In some cases, virtual exercise programs offered both real-time and pre-recorded options for participants, which was seen as the gold standard for some. As one participant described:

*The thing about recording is that you can choose many options...so that gives you more* var*iation... but the live one does provide a sense of community because I do happen to know the teacher and the people there. So, they both have advantages. (Female, 74 years old).*

Self-efficacy for technology not only facilitated older adults’ technology adoption but also increased with usage during the pandemic. Participants with prior technological experience and knowledge described a sense of confidence when using technology for home-based exercise participation. Increased technology usage also led to an enhanced sense of perceived competence among older adults who initially had lower technology literacy: “*I can do the basics and incrementally [my skills are] improving as I gain confidence as well.*” *(Female, 74 years old)*. Staying up to date with advancements in technology was also perceived as important to some, with one participant acknowledging the act of skill development as a cognitive benefit as well: “*When you get older, you have to find ways to use your brain. I think that learning technology will get the old brain waves going.*” *(Male, 64 years old)*. In contrast, a few older adults expressed hesitation in adopting technology due to privacy or security concerns. These concerns were often centered around the transmission of personal information to unknown sources. To mitigate the potential privacy risks, some older adults were particularly cautious when using technology or employed extra safety measures to further protect their privacy when using technology: “*After this [virtual interview], I’ll unplug my camera to my computer, just to be sure. I know I do not have to do that, but I will anyway.*” *(Male, 63 years old).*

Many participants were able to navigate technology independently, primarily by trial-and-error; however, some participants felt that lack of knowledge or prior experience with technology led to feelings of frustration. In particular, learning new technology on one’s own was perceived as an overwhelming pressure: “*you can get overloaded … all this stuff coming at me like, how to delete things or how to screen things…I still get frustrated because there’s so much I do not know.*” (*Female, 79 years old*). In these cases, the provision of technical support by older adults’ social networks, primarily through family members, played a crucial role in facilitating the use of technology for exercise classes and other applications. Peer support also positively influenced older adults’ technology usage: “*I just started talking to persons who had done it, and that’s how I learned how to do it… it’s either learn on your own or if you do not know, ask somebody who does know.*” (*Female, 60 years old*). Additionally, the availability of training or technical orientation sessions was suggested by some participants as a possible facilitator for technology adoption: “*It could have been helpful [to learn/use technology] if we could have had a demonstration.*” (*Female, 74 years old*).

## Discussion

Our findings highlight the significance of exercise knowledge and experiences, alongside adaptability in enabling and sustaining home-based physical activity during the pandemic. Older adults’ perceptions of their space, abilities, and safety appear to be as crucial as the factors themselves, influencing their willingness to adopt available remote physical activity supports and become active. Virtual exercise programs were well-received in our sample. Older adults preferred this format of support due to its flexibility, convenience, and accessibility. Furthermore, interactive virtual exercise classes with real-time supervision by exercise providers may be the most preferred for the social opportunity, and to ensure safety, especially for individuals who are new to exercise or have health conditions.

In our study, older adults with prior knowledge and experience often stayed physically active during the pandemic, motivated by the health and functional benefits. Their prior exercise experience gave them sufficient confidence and motivation to exercise independently at home. In prior studies, higher levels of exercise self-efficacy have been positively associated with increased physical activity levels, resulting in improvements in health outcomes such as enhanced muscular strength and improved aerobic endurance (44–48). Conversely, low self-efficacy has been linked to physical inactivity and sedentary behaviors (49–52).

Perceptions of a supportive home environment with adequate space and equipment facilitated physical activity during the pandemic. Comparison across interviews suggest that how older adults perceive their space, physical abilities, and safety is equally as significant as the factors themselves, ultimately shaping their engagement in home-based physical activity. For instance, a similar space (apartment/condominium) was described as both a facilitator and barrier to physical activity by different participants. Similarly, older adults’ perception of safety and their physical abilities influenced their willingness to participate in at-home physical activity. Some older adults felt confident while others reported feelings of uncertainty and fear of injury, leading to avoidance or reluctance to engage in exercise at home. Activity restriction due to a fear of falling has been reported in previous literature, highlighting fear of falling as a contributor to functional decline and loss of independence in older adults (53, 54). In these situations, trainer-led exercise programs, tailored to individual abilities and designed to boost balance confidence, could enhance feelings of safety and increase older adults’ engagement in physical activity at home (55–58).

More universally, access to outdoor spaces, especially during favorable weather, was identified as a key factor influencing physical activity during the pandemic. While winter weather acted as a deterrent, the presence of parks and trails in good weather provided valuable opportunities for outdoor activity, with walking being specifically noted as a preferred activity (59–61). Our findings echo other recent studies that emphasized the significance of the outdoor environment as an important avenue for supporting physical activities such as walking, running, and cycling during the COVID-19 pandemic (62, 63). Real-time supervision by exercise professionals appeared to be a crucial support for older adults with lower self-efficacy, knowledge, or skills for exercise. Our participants often sought supervision and guidance from exercise professionals and expressed that their physical activity was limited by the absence of trainer oversight. The virtual presence of exercise providers was valued as it instilled confidence and ensured the safety and appropriateness of exercise routines for many. In this regard, real-time virtual exercise programs where exercise professionals could observe and provide guidance were preferred as a desirable alternative to supervised onsite exercising (64). Previous research has also shown that supervised exercise programs lead to a decreased fear and incidence of adverse events (65, 66), improved health outcomes (e.g., balance and strength), and enhanced the overall effectiveness of the program (67).

Real-time virtual exercise classes also offer opportunities for interaction both with peers and exercise professionals. Our participants frequently highlighted that social engagement during real-time virtual programs served as a motivator and support for exercise. Virtual group exercise programs create an environment for companionship, encouragement, and connection with peers, just as it does with in-person exercise programs (68). The social aspect of exercise programs may be more important for older adults, given their higher risk of social isolation and loneliness (2), which can be further intensified during unpredictable situations such as ongoing social restrictions of a pandemic (69). Similar studies have also suggested that promoting meaningful social interaction can positively impact the physical and mental health of older adults (68, 70), particularly during quarantine periods (71, 72).

While technology access and skills have been cited as barriers to older adults’ adoption of virtual exercise programs (73–75), our participants expressed a positive attitude toward technology adoption. Many participants reported learning a new technology and/or increasing their usage of technology during the pandemic, providing a sense of accomplishment. Motivation to stay physically and mentally healthy were also described as factors that drove their adoption and usage of technology. Older adults have reported motivation to learn new technology for other purposes, including social media, online shopping, and telehealth (76–78). For some participants, support of family and friends was an important factor for technology adoption and usage, which is consistent with other research conducted among older adults, both prior to and during the COVID-19 pandemic (79–82). It is important to recognize that our sample most likely favored older adults who were technology-savvy, given the online administration of the survey. However, it is worth noting that other research that employed a combination of online and phone-based data collection methods also observed an increase in technology adoption and usage among older adults during the pandemic (83).

The importance of virtual exercise programs likely extends beyond a global pandemic, as virtual programs help overcome challenges faced by older adults in accessing in-person programs. This is particularly relevant for those living in rural areas or individuals with physical or transportation limitations. Offering virtual exercise programs can provide opportunities for physical activity to a broader range of older adults. Sustained virtual exercise programs also expand older adults’ access to trained exercise professionals within the comfort of their homes, making it easier for them to receive personalized exercise support. However, it is essential to recognize that internet access and speed limitations may continue to impede rural-dwelling older adults’ utilization of internet-based exercise programs at home (84). As high-speed internet availability gradually extends to various geographic regions, including rural and semi-rural areas in Canada (85), efforts to bridge the digital divide and advance internet infrastructure development across regions remain essential.

### Strengths and limitations

There are both strengths and limitations to this study. Our mixed-method approach enabled us to complement quantitative findings with qualitative insights, providing a comprehensive understanding of older adults’ experiences with at-home exercise during the pandemic and the underlying reasons influencing the adoption (or lack thereof) of remote supports for physical activity. The qualitative results provided rich and detailed information on how older adults engaged with virtual exercise and other remote physical activity programs, yielding valuable insights for the improvement of future home-based exercise programming. However, our findings should be interpreted with caution. Our sample is almost certainly biased toward individuals who were more technologically savvy and more physically active during the study period. The use of social media platforms for recruitment purposes introduces potential bias in our research, as social media users may be more comfortable with technology compared to those without social media. As such, this may not represent the broader older adult population due to challenges related to the digital divide and varying levels of technological literacy. Moreover, when utilizing social media platforms and employing snowball sampling, individuals sharing study information may introduce bias through selective dissemination. It is also important to note that our sample size was relatively small and predominantly consisted of Caucasian, highly educated, and mostly healthy older adults. This overrepresentation limits the generalizability of our findings to a broader population of older adults. Both the web-based survey and interviews were conducted in English, which could have resulted in the underrepresentation of older adults from other linguistic and cultural backgrounds. Furthermore, observed changes in physical activity may be influenced by recall bias when participants reflected on their pre-pandemic physical activity levels (86).

## Conclusion and implications

Despite the resumption of in-person physical activity programs in most communities, our findings underscore the potential benefit of virtual platforms to continue supporting physical activity and exercise programming in older adults’ homes, especially for those with limited access to in-person programming. Our study suggests that both real-time and pre-recorded virtual exercise supports can serve as viable alternatives, with sufficient technological capacity and support. Interactive real-time virtual exercise programs may be favored for the live interaction with exercise professionals and peers, while recorded exercise programs offer flexibility in timing. Further research is needed to establish best practices that ensure the safety and effectiveness of virtual exercise programming, promoting its long-term adoption for supporting a wider range of older adults remotely.

## Data availability statement

The quantitative dataset is available from the corresponding author upon reasonable request. However, the qualitative data, due to the highly personal nature of the responses and the risk of disclosure, are not publicly available. They can be obtained from the corresponding author upon reasonable request.

## Ethics statement

This study was approved by University of Waterloo Research Ethics Committee (#42191). This study was conducted in accordance with the local legislation and institutional requirements. All participants provided informed consent to participate in this study, consisting of online consent for the survey and verbal consent for interview participation.

## Author contributions

SM: Conceptualization, Formal analysis, Investigation, Methodology, Project administration, Software, Supervision, Validation, Visualization, Writing – original draft, Writing – review & editing, Data curation, Resources. SD: Conceptualization, Formal analysis, Investigation, Methodology, Project administration, Software, Validation, Visualization, Writing – original draft, Writing – review & editing. HD: Formal analysis, Project administration, Validation, Writing – original draft, Writing – review & editing. LM: Conceptualization, Formal analysis, Methodology, Resources, Supervision, Validation, Writing – original draft, Writing – review & editing.
